# Human Pressure Structures Range Contraction and Recovery Potential of the Snow Leopard

**DOI:** 10.1002/ece3.74217

**Published:** 2026-09-02

**Authors:** Erik Joaquín Torres‐Romero, Vincenzo Penteriani, William J. Ripple, James R. Allan, Adrian Treves, Kendall R. Jones, Rodney Jackson

**Affiliations:** ^1^ Ingeniería en Biotecnología‐ Universidad Politécnica de Puebla Puebla México; ^2^ Subdirección de Investigación y Posgrado, División de Biología Tecnológico Nacional de México Campus Zacapoaxtla Puebla México; ^3^ Department of Evolutionary Ecology, National Museum of Natural Sciences (MNCN) Spanish National Research Council (CSIC) Madrid Spain; ^4^ Department of Forest Ecosystems and Society Oregon State University Corvallis Oregon USA; ^5^ Conservation Biology Institute Corvallis Oregon USA; ^6^ Tropical Ice Safaris Ltd. Nairobi Kenya; ^7^ The Carnivore Coexistence Lab, Nelson Institute for Environmental Studies University of Wisconsin Madison Wisconsin USA; ^8^ Wildlife Conservation Society Global Conservation Program New York New York USA; ^9^ School of Agriculture, Food and Ecosystem Sciences, Faculty of Science The University of Melbourne Parkville Victoria Australia; ^10^ Snow Leopard Conservancy San Francisco California USA

**Keywords:** apex predator, conservation prioritization, human footprint, *Panthera uncia*, range contraction, spatial ecology

## Abstract

Human‐driven environmental change is rapidly altering species distributions worldwide, and anthropogenic activities are widely recognized as major drivers of range contraction and population decline. However, how human pressure interacts with other ecological processes to shape spatial patterns of persistence and recovery potential remains less well understood. Here, we present a spatially explicit framework integrating historical and current distributions with global human footprint data to quantify how human pressure influences range dynamics in the snow leopard ( *Panthera uncia* ). We classified the species' range into three states—lost, extant, and possibly extant—and evaluated their distribution across gradients of human footprint at multiple spatial resolutions. Our results indicate that range contraction was associated with elevated human pressure, with more than half of all lost areas (52.1%) occurring under high anthropogenic pressure. In contrast, extant populations were more frequently retained in lower‐pressure environments (53.4%) and occurred predominantly within the low and moderate disturbance classes, whereas possibly extant areas exhibited an intermediate but more disturbed profile, with nearly 35.3% of their area occurring under high or very high human pressure. These spatial patterns are consistent with the geographic distribution of human footprint, with higher disturbance concentrated in southern and eastern regions and lower‐impact areas persisting in northern and high‐elevation landscapes. Our findings demonstrate that human footprint exerts a consistent spatial filtering effect shaping species contraction, persistence, and recovery potential. By integrating historical baselines with contemporary spatial data, we show that current distributions likely reflect not only ecological suitability but also the legacy of long‐term anthropogenic impacts. This framework provides a scalable approach for conservation prioritization, emphasizing the need to limit human pressure in remaining refugia while targeting recovery in moderately impacted landscapes.

## Graphical abstract

We developed a spatially explicit framework to quantify how human pressure influences range dynamics in the snow leopard. Our results reveal a consistent spatial filtering effect, where human footprint shapes patterns of contraction, persistence, and recovery potential. This approach provides a scalable tool for prioritizing conservation in human‐modified landscapes.

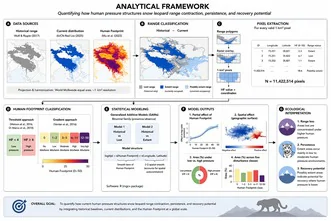

## 1 Introduction

Human‐driven environmental change is rapidly restructuring biodiversity worldwide, with extinction rates now exceeding background levels by orders of magnitude (Ceballos et al. 2015; IPBES 2019). Among terrestrial vertebrates, large carnivores are particularly vulnerable due to their extensive spatial requirements, low population densities, and sensitivity to human disturbance (Woodroffe 2000; Ripple et al. 2014). Severe range contractions have been documented across multiple large carnivores, including tigers (~95%), dholes (> 80%), snow leopards (> 77%), Asian black bears (> 60%), Sunda clouded leopards (> 50%), and sloth bears (> 38%) (Wolf and Ripple 2017). As a result, an increasing proportion of their historical distributions now overlap with areas of high human pressure, reflecting not only a reduction in available habitat but also a spatial reorganization in which remaining populations are increasingly confined to portions of the landscape with lower levels of anthropogenic disturbance (Di Marco et al. 2018; Venter et al. 2016; Allan et al. 2019; Jones et al. 2018).

Beyond the magnitude of these losses, the ecological consequences may be substantial. Large carnivores regulate trophic interactions and maintain ecosystem structure through top‐down processes (Estes et al. 2011; Ripple et al. 2014). Their loss—often termed trophic downgrading—has been linked to cascading effects, including altered herbivore dynamics, vegetation structure, and ecosystem functioning across multiple biomes (Estes et al. 2011; Leighton et al. 2023). Although human activities are well established as major drivers of range contraction and population decline (Ripple et al. 2014; Torres‐Romero et al. 2020, 2025), how anthropogenic pressure interacts with other ecological processes to shape broad‐scale patterns of persistence and recovery potential remains less well understood, particularly where historical baselines are unavailable or cannot be delineated with a high degree of confidence.

Current global assessments have documented strong spatial associations between human pressure and biodiversity loss (Venter et al. 2016; Di Marco et al. 2018; Torres‐Romero et al. 2020, 2025). However, they remain limited in their ability to integrate historical distributions, present‐day occurrence, and anthropogenic pressure within a unified spatial framework. Most studies focus on contemporary distributions, often without quantifying where range loss has occurred relative to historical baseline (Venter et al. 2016; Di Marco et al. 2018; Torres‐Romero et al. 2020, 2025). Without this temporal context, current distributions may be misinterpreted as reflecting environmental suitability rather than the legacy of anthropogenic filtering. Furthermore, the extent to which areas of potential recovery remain viable under current levels of human pressure remains poorly understood and often uncertain, limiting the development of robust or possibly even effective restoration strategies.

A more vigorous way to address these limitations is through spatially explicit metrics of cumulative human influence, such as the Human Footprint index, which integrates land transformation, infrastructure, and accessibility into a globally consistent measure of anthropogenic pressure (Venter et al. 2016; Mu et al. 2022). Thresholds in human footprint values (e.g., ≥ 4; see Methods) have been linked to ecological transitions between relatively intact and human‐dominated systems. These thresholds are also associated with elevated extinction risk and habitat degradation (Di Marco et al. 2018; Watson et al. 2016). Despite its widespread use in biodiversity assessments, this framework has rarely been applied to explicitly link past range contraction, present distribution, and future recovery potential within a unified spatial context, particularly for species inhabiting remote and topographically complex mountain systems.

The snow leopard ( *Panthera uncia* ) provides a particularly informative model species to address how human pressure is associated with patterns of range contraction, persistence, and recovery potential. Distributed across the mountainous regions of Central and South Asia, this species occupies ecosystems that have historically been characterized by low human density, high environmental heterogeneity, and strong topographic and climatic constraints (McCarthy and Mallon 2016). However, rapid expansion of human activities—including infrastructure development, livestock grazing, and resource extraction—is increasingly reshaping these landscapes through indirect pathways, increasing human accessibility and altering habitat structure, prey availability, and landscape connectivity (Li et al. 2014; McCarthy and Mallon 2016; Heiner et al. 2016). These changes ultimately affect the demographic processes underlying population persistence, including survival and recruitment. Importantly, several key correlates of population dynamics, such as human–wildlife conflict and prey depletion, are not well captured by global datasets (McCarthy and Mallon 2016). As a result, linking large‐scale indicators of human pressure with underlying ecological mechanisms presents a major challenge. Human‐driven landscape changes are not only reducing suitable habitat but also fragmenting populations and modifying the spatial configuration of ecological processes across the species' range. Despite its widespread use in biodiversity assessments, this framework has rarely been applied to explicitly link past range contraction, present distribution, and future recovery potential within a unified spatial context, particularly for species inhabiting remote and topographically complex mountain systems like snow leopards.

Here, we develop a spatially explicit assessment of snow leopard range dynamics by integrating broad historical distribution data (Wolf and Ripple 2017) and current distribution data (IUCN 2025) with the recently updated Human Footprint dataset (Mu et al. 2022). We quantify three complementary range states—lost, extant, and possibly extant—and evaluate their distribution across gradients of human pressure. By explicitly distinguishing between these range states, we assess whether human pressure—as a composite measure of anthropogenic influence including infrastructure, land use, and human accessibility captured by the Human Footprint index—acts as a consistent spatial filter across temporal dimensions of species distribution. This framework allows us to test whether areas of historical loss are disproportionately associated with high current human impact, while extant populations are more frequently associated with lower gradients of current human pressure, and whether possibly extant areas occur across intermediate levels of disturbance.

## 2 Materials and Methods

**FIGURE 1:**
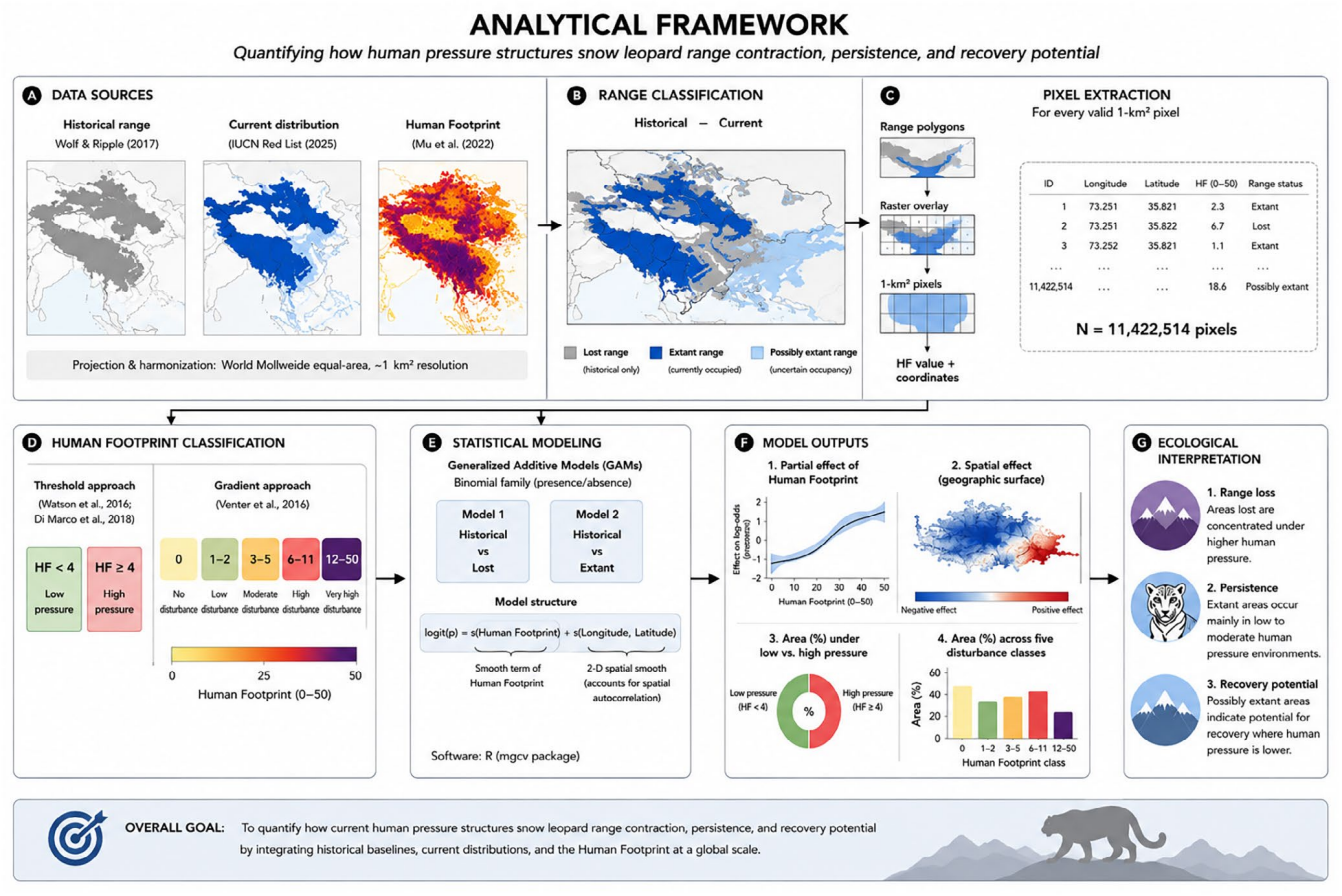
Analytical framework of the study. Historical range estimates (Wolf and Ripple 2017), current distribution data (IUCN Red List, 2025), and the global Human Footprint dataset (Mu et al. 2022) were harmonized to a common equal‐area projection (~1‐km^2^ resolution). Historical and current distributions were integrated to classify the landscape into lost, extant, and possibly extant range categories. Human Footprint values were extracted for every valid 1‐km^2^ pixel (*n* = 11,422,514) and analyzed using complementary threshold‐based (< 4 vs. ≥ 4) and five‐level disturbance classifications. Generalized additive models (GAMs) were then used to evaluate the relationship between Human Footprint and range dynamics while accounting for spatial autocorrelation. The framework summarizes the complete analytical pipeline used throughout the study. This figure is a conceptual representation of the analytical workflow. Maps, diagrams, and graphical elements are schematic and are intended solely to illustrate the sequence of spatial processing and statistical analyses. They are not presented as exact cartographic products, parameter estimates, or model predictions. Quantitative results and human impact map are presented in Figures 2, 3, 4, Figures S1, and the Results section.

We compiled spatial data on the distribution of the snow leopard from two complementary sources representing different temporal baselines. Current distribution data were obtained from the IUCN Red List of Threatened Species (https://www.iucnredlist.org; accessed March 2025). Historical distribution data were derived from Wolf and Ripple (2017), representing reconstructed historical ranges prior to major anthropogenic contraction and based on an updated version of the global large carnivore range dataset originally developed by Morrison et al. (2007). Because of limited empirical data on the species, historical range estimates should be interpreted as broad approximations rather than precise reconstructions, and may include areas of marginal or unsuitable habitat due to coarse spatial delineation. Together, these datasets allowed us to distinguish broad‐scale spatial contrasts between the reconstructed historical distribution and the current resident distribution, thereby identifying inferred areas of persistence and range loss across the species' geographic distribution while ensuring consistency with widely used global baselines of large carnivore range contraction. The overall analytical workflow is summarized in Figure 1.

To quantify anthropogenic pressure, we used the global Human Footprint dataset (Mu et al. 2022). This dataset integrates eight variables capturing key dimensions of human activity, including built environments, human population density, electric infrastructure, croplands, pasture lands, roads, railways, and navigable waterways. Each variable is standardized and combined to produce a cumulative Human Footprint score ranging from 0 to 50 at a spatial resolution of approximately 1 km^2^. This index has been widely applied in global biodiversity assessments and has been shown to be strongly associated with patterns of species decline, habitat loss, and extinction risk (Venter et al. 2016; Di Marco et al. 2018; Torres‐Romero et al. 2025). Accordingly, in this study, Human Footprint is interpreted as a proxy for accessibility and cumulative human influence at broad spatial scales, rather than a direct measure of all ecological pressures affecting the species. Because the dataset is designed for broad‐scale analyses, it may not fully capture fine‐scale variation in anthropogenic pressure across rugged mountain landscapes. Consequently, our analyses are intended to characterize regional patterns of human influence rather than local habitat conditions.

All spatial analyses were conducted in ArcGIS 10.1 (ESRI 2011) using an equal‐area World Mollweide projection to ensure accurate and comparable area estimates across spatial layers. ArcGIS was used exclusively for standard geospatial preprocessing operations (projection, overlay, geometric difference, and raster extraction), whereas all statistical analyses were performed independently in R. All datasets were first harmonized to a common projection, spatial resolution, and extent prior to analysis. As summarized in Figure 1, we then overlaid historical and current distribution layers with the Human Footprint raster to extract spatially explicit information on human pressure across the species' range. Range dynamics were quantified by defining three complementary spatial categories: (i) lost range, representing areas included in the historical distribution but absent from the current distribution; (ii) extant range, representing areas currently occupied by the species; and (iii) potential (or possibly extant) range, representing areas where presence is uncertain and suitable conditions may persist but occupancy is not consistently confirmed. The lost range was derived using a geometric difference operation between historical and current distributions, while extant and possibly extant ranges were directly obtained from IUCN spatial classifications. For each range category, we calculated total area (km^2^) and extracted the distribution of Human Footprint values at the pixel level. Human Footprint values were extracted from every valid 1‐km^2^ pixel contained within each range category, resulting in a database of 11,422,514 observations. Each pixel retained its geographic coordinates and Human Footprint value, allowing analyses to be conducted using the complete dataset rather than a subsample.

**FIGURE 2:**
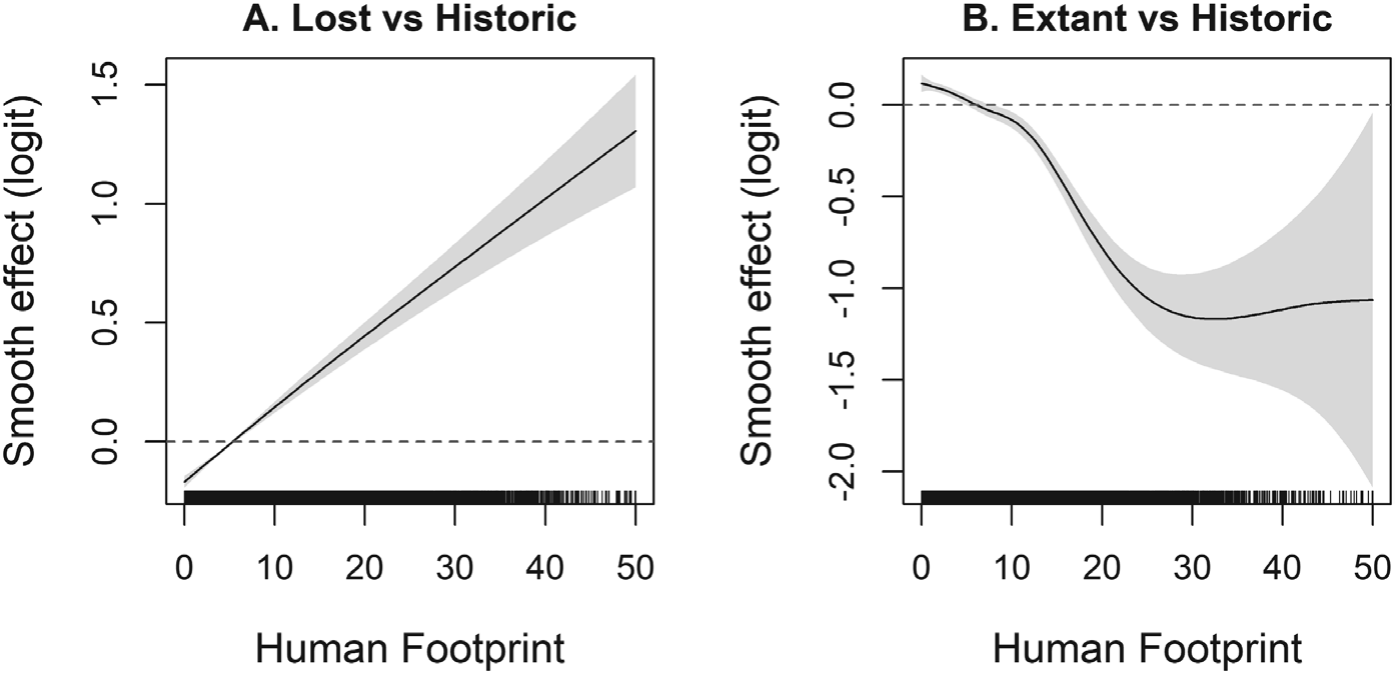
Generalized additive model (GAM) smooth functions describing the relationship between Human Footprint and snow leopard range dynamics after accounting for spatial autocorrelation. Panel A shows the smooth effect of Human Footprint on the probability of range loss relative to the historical distribution, whereas Panel B shows the corresponding relationship for extant versus historical range. Gray shading represents the 95% confidence interval around the fitted smooth, and rug marks indicate the distribution of Human Footprint observations.

To evaluate whether anthropogenic pressure explained range dynamics while accounting for spatial dependence, we fitted generalized additive models (GAMs) with a binomial error distribution using the *mgcv* package (Wood and Wood 2015) in R. Two independent models were fitted using the historical distribution as the reference condition: (i) lost versus historical range and (ii) extant versus historical range. In both models, Human Footprint was included as a smooth term, whereas geographic coordinates were incorporated as a two‐dimensional smooth surface to account explicitly for spatial autocorrelation. Model performance was evaluated using approximate significance tests of smooth terms and deviance explained. The fitted smooth functions describing the relationship between Human Footprint and range dynamics are presented (Figure 2).

Human Footprint values were classified using two complementary approaches to evaluate how anthropogenic pressure structures range extent dynamics. First, we applied a threshold‐based classification to distinguish between low and high human pressure, following previous studies (Watson et al. 2016; Di Marco et al. 2018). Specifically, areas with Human Footprint values < 4 were defined as low‐pressure systems, whereas values ≥ 4 were considered high‐pressure systems, corresponding to landscapes where human influence is sufficiently intense to substantially alter ecosystem structure and increase extinction risk. Second, we applied a finer categorical classification following Venter et al. (2016) to capture gradients of disturbance intensity, defining five levels: no disturbance (0), low disturbance (1–2), moderate disturbance (3–5), high disturbance (6–11), and very high disturbance (12–50). This dual classification framework allowed us to assess both broad thresholds and finer‐scale gradients of human impact across the species' range, thereby reducing potential bias associated with single‐threshold approaches and providing a more realistic representation of disturbance variation. Finally, for each range category we quantified total area (km^2^) and the proportional distribution of area across both threshold‐based (low vs. high) and categorical disturbance classes. All area calculations were performed in an equal‐area projection to ensure consistency and avoid spatial bias. This approach enabled a direct evaluation of whether human pressure is associated with a spatial filtering effect structuring patterns of historical range loss, current persistence, and potential recovery across the snow leopard's distribution. This analysis assumes that (i) available historical and current range maps provide a reasonable approximation of large‐scale distribution patterns, and (ii) Human Footprint values capture broad spatial variation in anthropogenic pressure relevant to species persistence, despite not explicitly representing all underlying ecological mechanisms.

## 3 Results

**FIGURE 3:**
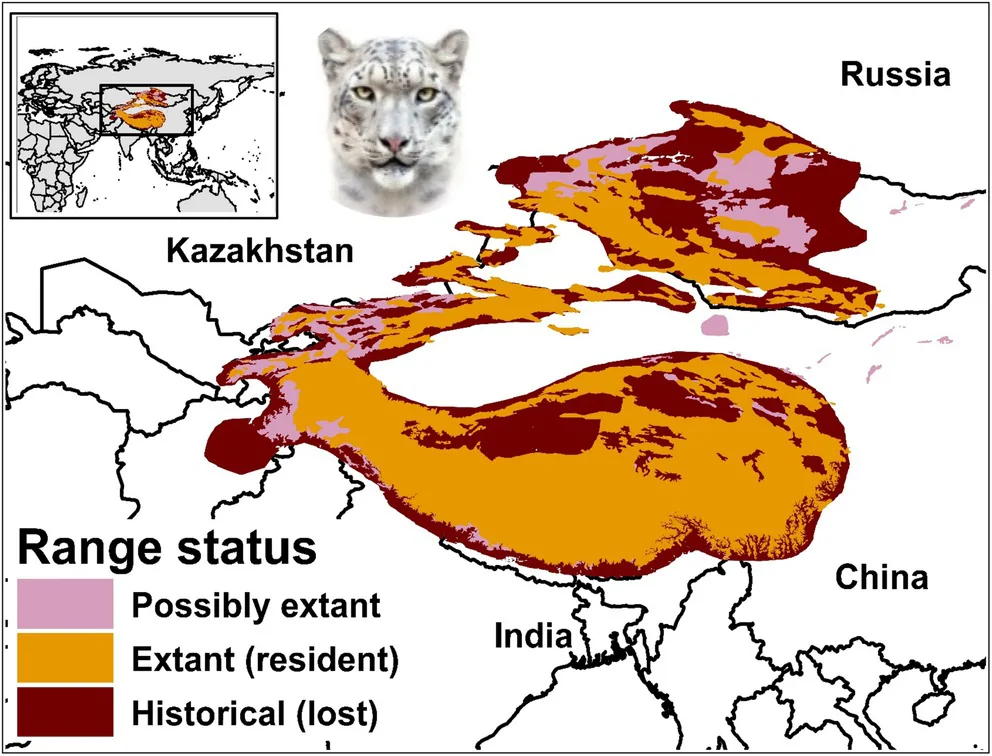
Spatial distribution of the historical, extant, and possibly extant range of the snow leopard. Geographic distribution of the snow leopard ( *Panthera uncia* ) across Central and South Asia showing extant resident range (orange), possibly extant areas with uncertain or intermittent presence (light pink), and historically occupied areas that have been lost (dark red). Historical lost range was derived as the difference between reconstructed historical distribution (Wolf and Ripple 2017) and the current IUCN distribution, representing areas inferred to have been occupied prior to major anthropogenic contraction but no longer included within the species' current range. Extant and possibly extant distributions follow the current IUCN classifications.

We found that the spatial distribution of historical, extant, and possibly extant range states exhibits clear geographic structuring across the species' range (Figure 3). Extant populations are primarily concentrated within a continuous belt across the central and southern portions of the range, whereas historically occupied areas are more fragmented and disproportionately distributed along peripheral and northern regions. We also found that areas classified as possibly extant occur as discontinuous patches that frequently connect extant and historically lost regions. Overall, current populations occupy a reduced and spatially structured subset of the broader historical distribution.

**FIGURE 4:**
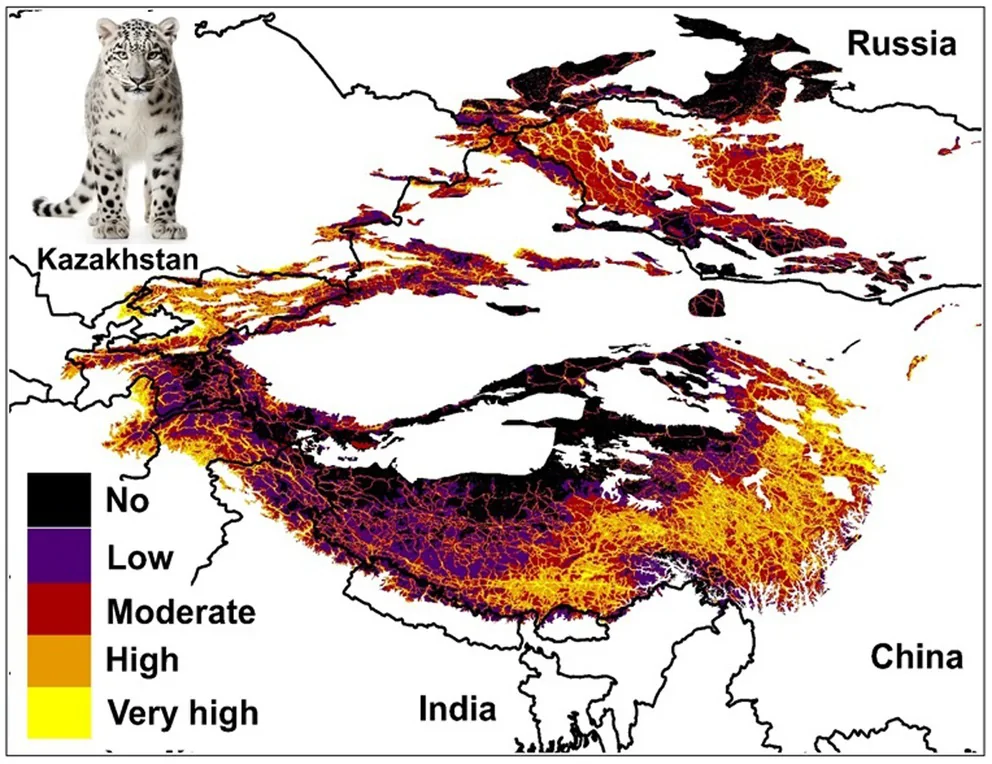
Spatial heterogeneity in anthropogenic pressure across the extant and possibly extant snow leopard range. Spatial distribution of the five Human Footprint disturbance classes across snow leopard ( *Panthera uncia* ) distribution. Human Footprint values were classified into five categories: No disturbance (0), low (1–2), moderate (3–5), high (6–11), and very high (12–50), following Venter et al. (2016). Notably, the influence of road networks, which link human settlements (e.g., villages, towns, cities) along with large‐scale infrastructure development is clearly evident.

Human Footprint exhibited pronounced spatial heterogeneity across the snow leopard's distribution (Figure 4). High and very high Human Footprint levels were concentrated primarily along the southern and eastern portions of the species' range, whereas no and low disturbance predominated across many northern and high‐elevation regions. Moderate disturbance formed extensive transition zones between these contrasting areas, generating a continuous spatial gradient of anthropogenic pressure throughout the species' distribution (Figure 4).

**FIGURE 5:**
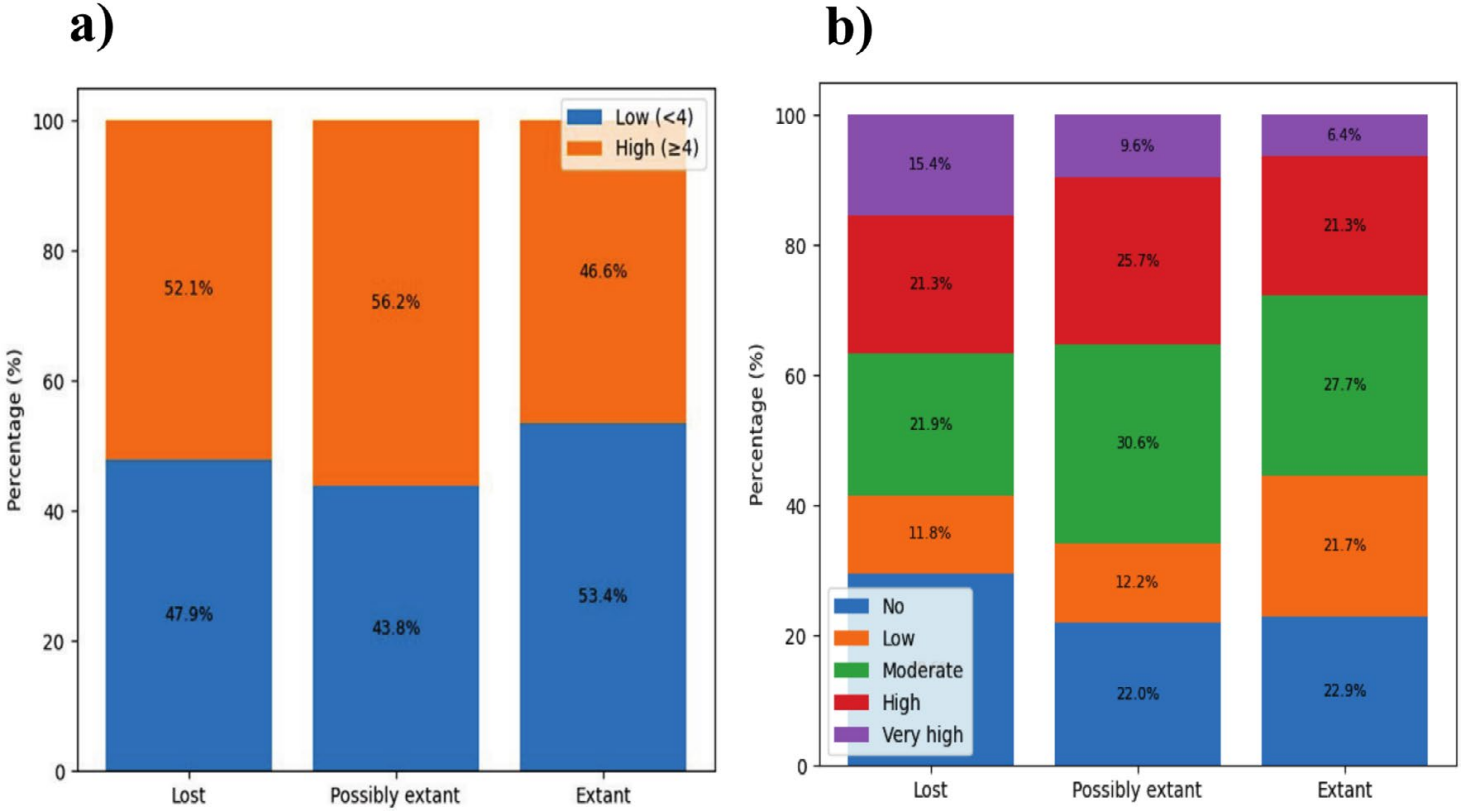
Distribution of human pressure across snow leopard range states. (a) Proportion of lost, extant, and possibly extant areas under low (< 4) and high (≥ 4) human pressure based on the Human Footprint index. Lost areas show a higher proportion of high‐pressure conditions, whereas extant areas are more represented under low‐pressure conditions. Possibly extant areas exhibit intermediate patterns. (b) Distribution of range states across five levels of human disturbance: No disturbance (0), low (1–2), moderate (3–5), high (6–11), and very high (12–50). Lost areas are more strongly represented in moderate to very high disturbance classes, whereas extant areas are more associated with low to moderate disturbance. Possibly extant areas show intermediate distributions across disturbance levels.

Using the complete Human Footprint raster (11,422,514 valid 1‐km^2^ pixels), we found marked differences in anthropogenic pressure among range states (Figures 2 and 5a and Figure S1). Relative to the historical distribution, lost areas contained a greater proportion of pixels exposed to high human pressure (52.1%), whereas extant areas were dominated by low human pressure (53.4%). Possibly extant areas exhibited the highest proportion of high human pressure (56.2%), indicating that many areas where occupancy remains uncertain already occur within heavily modified landscapes (Figure 5a).

Across the five Human Footprint classes, historical range exhibited a relatively balanced distribution among disturbance levels. In contrast, lost areas showed a marked increase in the proportion of very highly disturbed landscapes (15.4%), whereas extant populations were disproportionately associated with low (21.7%) and moderate (27.7%) disturbance and showed the lowest representation within very highly disturbed landscapes (6.4%). Possibly extant areas displayed an intermediate but more disturbed pattern, with nearly 35.3% of their area occurring under high or very high human pressure (Figure 5b). The complete distributions of Human Footprint values across historical, lost, extant, and possibly extant range states are shown in Figure S1. Despite substantial overlap among distributions, differences in their central tendency are consistent with the categorical patterns observed in Figure 5.

Generalized additive models that explicitly accounted for spatial autocorrelation confirmed that Human Footprint remained a highly significant predictor of snow leopard range dynamics (both models, *p* < 0.001, Figure 2). In the comparison between lost and historical distributions, Human Footprint significantly explained extirpation probability (edf = 1.40, *χ* ^2^ = 280.1), while spatial location also accounted for substantial variation (edf = 96.7, *χ* ^2^ = 5721.0; deviance explained = 13.1%). Likewise, comparisons between extant and historical distributions showed that Human Footprint significantly predicted current persistence (edf = 5.50, *χ* ^2^ = 302.7), together with a strong spatial component (edf = 96.1, *χ* ^2^ = 4575.1; deviance explained = 10.0%).

## 4 Discussion

Our results reveal a clear and spatially structured association between human pressure and range dynamics in the snow leopard, with range contraction closely linked to gradients of anthropogenic disturbance and displaying a non‐random spatial pattern across the landscape. By explicitly integrating historical, extant, and possibly extant distributions, we found that areas classified as lost are disproportionately concentrated in regions characterized by higher levels of human pressure, whereas extant populations are more frequently retained in comparatively lower‐moderate pressure environments. Thus, human pressure appears to function as a large‐scale spatial filter structuring the persistence of snow leopards, as also observed in other wide‐ranging carnivore species (Ripple et al. 2014; Torres‐Romero et al. 2020, 2025). Notably, this filtering effect was consistently evident across multiple analytical resolutions, including both a threshold‐based classification (low vs. high pressure; Figure 5a) and a finer categorical gradient (five disturbance levels; Figure 5b). Although the five‐category classification illustrates the full gradient of human pressure, statistical inference was based on the previously established Human Footprint threshold (< 4 vs. ≥ 4) proposed by Watson et al. (2016) and Di Marco et al. (2018), which has been associated with ecological transitions and increased species extinction risk. Because our analysis is based on complete spatial coverage of the species' distribution rather than a sampled subset, the patterns reported here reflect underlying spatial structure rather than artifacts of sampling‐based statistical inference (Franklin 2010). Our results show that descriptive patterns were independently supported by generalized additive models that explicitly accounted for spatial autocorrelation (Figure 2). Even after controlling for geographic structure, Human Footprint remained a highly significant predictor of both range loss and current persistence, indicating that the observed relationships cannot be explained solely by the spatial clustering of environmental conditions or sampling density.

These findings are consistent with a growing body of global evidence identifying human activities, especially large‐scale developments or activities responsible for severe habitat degradation, as a major cause of large carnivore declines and range contractions (Woodroffe 2000; Ripple et al. 2014; Di Marco et al. 2018). However, our study advances beyond previous work by explicitly incorporating historical baselines and quantifying how range loss, persistence, and recovery potential are spatially structured along gradients of current human pressure. Previous global assessments have documented extensive contractions in large carnivores (Morrison et al. 2007; Wolf and Ripple 2017), yet have rarely resolved how such contractions are distributed relative to anthropogenic pressure across landscapes. Here, by linking historical and current distributions within a unified spatial framework, we show that contemporary patterns of occurrence may not be fully explained by environmental suitability alone but instead represent the combined outcome of ecological constraints and long‐term anthropogenic filtering inferred from the latest Human Footprint dataset (Mu et al. 2022).

Although widely used as consistent global baselines for large carnivore range contraction, historical reconstructions are necessarily coarse and may include unsuitable habitats. Consequently, the maps presented here should be interpreted as broad‐scale spatial representations of inferred range contraction derived from differences between historical and current distributions, rather than as precise reconstructions of documented local extirpations. Accordingly, estimates of range loss represent approximations rather than exact measures of historical occupancy. Similarly, although the Human Footprint dataset provides a globally consistent measure of anthropogenic pressure, its approximately 1‐km^2^ spatial resolution may not fully capture fine‐scale variation in human influence across rugged mountain landscapes. Consequently, our analyses are intended to characterize broad‐scale regional patterns rather than local habitat conditions. When contrasted with spatial models of ecological suitability (e.g., McCarthy and Mallon 2016), our results reveal a pronounced mismatch between potential habitat and realized distribution, indicating that substantial portions of climatically and ecologically suitable range are currently not occupied and may be partly constrained by human pressure. This spatial decoupling indicates that anthropogenic disturbance may contribute to shaping the functional limits of the species' range, alongside underlying environmental constraints, rather than acting as the sole determinant of where populations can persist. This distinction is critical, as failure to account for historical range loss may lead to systematic underestimation of the role of human pressure in shaping present‐day biodiversity patterns (Venter et al. 2016; Allan et al. 2019; Torres‐Romero et al. 2020, 2025). Consistent with this, current distributions represent a spatially constrained subset of the species' fundamental niche (Hutchinson 1957; Soberón 2007), shaped by persistent and current human influence rather than ecological limits alone. Because the Human Footprint index integrates multiple long‐term components of human infrastructure and land use, current Human Footprint values likely reflect the cumulative effects of anthropogenic pressures through time, allowing broad‐scale inference regarding historical range contraction. An additional mechanism that may contribute to the observed mismatch between human pressure and current occupancy is source–sink dynamics (Frans and Liu 2024; Craig et al. 2025). Some snow leopard populations may persist despite relatively high human pressure through demographic support from neighboring source populations located in less disturbed areas (Pulliam 1988; Runge et al. 2006). Consequently, present‐day occupancy may not always reflect local anthropogenic pressure alone but also regional population connectivity and dispersal processes. Although evaluating source–sink dynamics requires demographic and movement data that are currently unavailable across the species' entire distribution, this mechanism could partially contribute to the observed spatial patterns. Future studies integrating demographic monitoring, movement ecology, and landscape connectivity will be essential to explicitly evaluate this hypothesis. However, the persistence of the Human Footprint effect after explicitly accounting for spatial autocorrelation indicates that source–sink dynamics alone are unlikely to explain the broad‐scale spatial patterns observed in this study.

Nevertheless, we acknowledge that the Human Footprint index does not directly capture: (a) key influences on snow leopard population dynamics, including prey depletion, human–wildlife conflict, and localized hunting pressures; (b) some globally recognized drivers such as climate change, pollution, infectious diseases, overexploitation, invasive species, and illegal killing; and (c) increases in livestock numbers and the expansion and intensification of pastoral systems across parts of the species' range, which may further amplify human–wildlife interactions, including competition for forage, prey depletion, and retaliatory killing across the snow leopard range (IPBES 2019; Maxwell et al. 2016; Symes et al. 2018). Despite these limitations on our inferences, the consistency of patterns across multiple analytical approaches suggests that the observed relationships capture robust large‐scale structure in the interaction between human pressure and species distribution. However, although differences among range categories are moderate, they are consistent and directionally robust across analytical approaches, indicating that human pressure should be interpreted as one of multiple interacting factors influencing range dynamics rather than as a sole or dominant cause.

As a result, our estimates likely represent a conservative approximation of the total anthropogenic pressure experienced across the species' range, suggesting that the true constraints on population persistence may be stronger or more complex than those depicted in our analyses. A key finding of our analysis is that extant populations are not restricted to areas of minimal human disturbance but instead persist across a gradient of pressure, including landscapes characterized by moderate levels of anthropogenic influence (Figure 4). This pattern indicates a measurable degree of ecological and behavioral tolerance to human‐modified environments, consistent with previous studies documenting the capacity of snow leopards to persist in multi‐use landscapes under specific socioecological conditions, particularly where human tolerance, prey availability, and effective non‐lethal livestock management practices are maintained (McCarthy and Mallon 2016; Li et al. 2014). However, the tolerances of snow leopards for human pressures are clearly bounded. The reduced representation of extant areas in high and very high disturbance categories, together with the higher prevalence of lost areas within these classes, is consistent with the possibility of thresholds beyond which persistence becomes increasingly unlikely. These results align with previous findings showing that human footprint values above key thresholds (e.g., ≥ 4; Figure 5a) are consistently associated with habitat degradation, fragmentation, and elevated extinction risk (Venter et al. 2016; Watson et al. 2016; Di Marco et al. 2018; Torres‐Romero et al. 2025). Therefore, our findings reinforce the concept that human pressure may impose nonlinear constraints on species persistence.

The intermediate patterns observed in possibly extant areas provide further insight into how current human pressure may constrain future recovery potential. These areas are disproportionately associated with moderate and high disturbance categories (Figure 5b), suggesting two alternative explanations. First, these areas may represent landscapes where ecological conditions remain partially suitable but where recent increases in human pressure may now limit stable occupancy. Alternatively, human pressure in these areas may have been long‐standing, and other unmeasured ecological or social factors may have previously allowed persistence until recent local extirpation. Distinguishing between these alternatives would require temporal data on changes in human pressure, with important implications for conservation and restoration strategies. Notably, the relatively high levels of human pressure observed in possibly extant areas suggest that these regions should not be interpreted as uniformly recoverable, but rather as heterogeneous transitional zones where persistence may depend on local ecological conditions, human tolerance, and management context.

At broader spatial scales, our results demonstrate that human pressure is pervasive across much of the snow leopard's range, including areas where populations continue to persist (Figure 4). This pattern is consistent with evidence that even historically remote high‐elevation environments are increasingly affected by infrastructure development, resource extraction, and expanding pastoral systems, reducing their function as effective refugia for the species (Heiner et al. 2016; McCarthy and Mallon 2016). Moreover, human–wildlife conflict, particularly associated with predation on livestock, represents a key mechanism linking human presence to population declines and local extirpations across the species' range (McCarthy and Mallon 2016). This widespread overlap underscores the growing importance of strategies that enable coexistence between wildlife and human activities. At the same time, the concentration of extant populations in relatively lower‐pressure environments highlights the continued importance of the remaining intact or semi‐intact landscapes as critical refugia. These areas likely function as population strongholds, maintaining core ecological processes and serving as sources for dispersal and recolonization.

On the basis of our results, management planning should adopt a dual strategy that (i) prioritizes the protection of low‐pressure refugia to prevent further contraction, and (ii) promotes management interventions in moderately impacted landscapes to enhance recovery potential and connectivity. Such an approach is consistent with global conservation frameworks emphasizing the need to both retain intact ecosystems and restore degraded landscapes to halt biodiversity loss (IPBES 2019; Watson et al. 2016; Torres‐Romero et al. 2025). By integrating historical baselines with contemporary spatial data, we provide a more complete understanding of how anthropogenic pressures restructure species distributions across time. Given that similar pressures operate across ecosystems worldwide, this framework is broadly applicable for identifying priority areas for management, protection, and restoration. In an increasingly human‐modified world, recognizing and managing the spatial constraints imposed by human pressure will be essential for preventing further range contraction and enabling the long‐term persistence of wide‐ranging species.

Importantly, the consistency between the descriptive analyses (Figures 4 and 5), the spatially explicit generalized additive models and violin plots (Figure 2 and Figure S1) strengthens the evidence that elevated Human Footprint values are associated with increased range loss and reduced persistence across the species' distribution. This concordance across complementary analytical approaches provides a robust quantitative basis for identifying spatial thresholds that can inform conservation prioritization. These results emphasize the urgent need to prevent further expansion of human pressure into remaining low‐impact areas while simultaneously targeting moderately disturbed landscapes where recovery remains more feasible. Accordingly, effective management strategies should move beyond static protection frameworks and instead incorporate dynamic, spatially explicit approaches that address both refugia and human‐dominated systems. More broadly, our findings demonstrate that anthropogenic pressure is not simply a correlate of biodiversity loss but a key factor associated with the large‐scale restructuring of species persistence patterns. As anthropogenic influence continues to expand, failure to explicitly incorporate these constraints into planning may result in continued range contraction and diminished recovery potential across wide‐ranging species. Conversely, integrating spatial thresholds of human pressure into management strategies provides a potentially scalable and actionable pathway to mitigate biodiversity loss and promote long‐term species persistence, contributing to a more realistic understanding of the spatial limits within which large carnivores—and biodiversity more broadly—can persist in an increasingly human‐dominated world.

## Author Contributions

**Erik Joaquín Torres‐Romero:** conceptualization (equal), data curation (equal), formal analysis (equal), investigation (equal), methodology (equal), supervision (equal), validation (equal), visualization (equal), writing – original draft (equal), writing – review and editing (equal). **Vincenzo Penteriani:** writing – review and editing (equal). **William J. Ripple:** writing – review and editing (equal). **James R. Allan:** writing – review and editing (equal). **Adrian Treves:** writing – review and editing (equal). **Kendall R. Jones:** writing – review and editing (equal). **Rodney Jackson:** funding acquisition (equal), writing – review and editing (equal).

## Conflicts of Interest

The authors declare no conflicts of interest.

## Supporting information


**Figure S1:** Distribution of Human Footprint values across historical, lost, extant and possibly extant range states. Violin plots illustrate the full distribution of Human Footprint values, whereas embedded boxplots show the median and interquartile range.

## Data Availability

All primary spatial datasets used in this study are publicly available from the IUCN Red List (iucnredlist.org), Wolf and Ripple (2017), and Mu et al. (2022). The compiled data points (Human Footprint values) and categorical classifications used for the statistical analyses are available as Supporting Information accompanying this article.

## References

[ece374217-bib-0001] Allan, J. R. , J. E. Watson , M. Di Marco , et al. 2019. “Hotspots of Human Impact on Threatened Terrestrial Vertebrates.” PLoS Biology 17, no. 3: e3000158. 30860989 10.1371/journal.pbio.3000158 PMC6413901

[ece374217-bib-0004] Ceballos, G. , P. R. Ehrlich , A. D. Barnosky , A. García , R. M. Pringle , and T. M. Palmer . 2015. “Accelerated Modern Human–Induced Species Losses: Entering the Sixth Mass Extinction.” Science Advances 1, no. 5: e1400253. 26601195 10.1126/sciadv.1400253 PMC4640606

[ece374217-bib-0006] Craig, E. F. , M. Szojka , R. M. Germain , and L. G. Shoemaker . 2025. “Species Occupancy Is Inflated by Sink Populations in Productive Environments but Not Unproductive Environments.” Ecology 106, no. 4: e70089. 40265302 10.1002/ecy.70089 PMC12015658

[ece374217-bib-0008] Di Marco, M. , O. Venter , H. P. Possingham , and J. E. Watson . 2018. “Changes in Human Footprint Drive Changes in Species Extinction Risk.” Nature Communications 9, no. 1: 4621. 10.1038/s41467-018-07049-5 PMC6218474 30397204

[ece374217-bib-0009] ESRI . 2011. ArcGIS Desktop: Release 10.1. Environmental Systems Research Institute.

[ece374217-bib-0010] Estes, J. A. , J. Terborgh , J. S. Brashares , et al. 2011. “Trophic Downgrading of Planet Earth.” Science 333, no. 6040: 301–306. 21764740 10.1126/science.1205106

[ece374217-bib-0011] Franklin, J. 2010. Mapping Species Distributions: Spatial Inference and Prediction. Cambridge University Press.

[ece374217-bib-0012] Frans, V. F. , and J. Liu . 2024. “Gaps and Opportunities in Modelling Human Influence on Species Distributions in the Anthropocene.” Nature Ecology & Evolution 8, no. 7: 1365–1377. 38867092 10.1038/s41559-024-02435-3 PMC11239511

[ece374217-bib-0013] Heiner, M. , J. Oakleaf , G. Davaa , B. Yundon , and J. Kiesecker . 2016. “Emerging Threats to Snow Leopards From Energy and Mineral Development.” In Snow Leopards: Biodiversity of the World: Conservation From Genes to Landscapes, edited by T. McCarthy and D. Mallon , 1st ed., 116–122. Elsevier.

[ece374217-bib-0014] Hutchinson, G. E. 1957. “Concluding Remarks.” Cold Spring Harbor Symposia on Quantitative Biology 22: 415–427.

[ece374217-bib-0015] IPBES . 2019. “Intergovernmental Science‐Policy Platform on Biodiversity and Ecosystem Services.” *Summary for Policy Makers of the Global Assessment Report on Biodiversity and Ecosystem Services of the Intergovernmental Science‐Policy Platform on Biodiversity and Ecosystem Services*. IPBES Secretariat.

[ece374217-bib-0016] IUCN . 2025. “The IUCN Red List of Threatened Species.” https://iucnredlist.org.

[ece374217-bib-0017] Jones, K. R. , O. Venter , R. A. Fuller , et al. 2018. “One‐Third of Global Protected Land Is Under Intense Human Pressure.” Science 360, no. 6390: 788–791. 29773750 10.1126/science.aap9565

[ece374217-bib-0018] Leighton, G. R. , W. Froneman , L. E. Serieys , and J. M. Bishop . 2023. “Trophic Downgrading of an Adaptable Carnivore in an Urbanising Landscape.” Scientific Reports 13, no. 1: 21,582. 38062237 10.1038/s41598-023-48868-x PMC10703923

[ece374217-bib-0019] Li, J. , D. Wang , H. Yin , et al. 2014. “Role of Tibetan Buddhist Monasteries in Snow Leopard Conservation.” Conservation Biology 28, no. 1: 87–94. 23992599 10.1111/cobi.12135

[ece374217-bib-0020] Maxwell, S. L. , R. A. Fuller , T. M. Brooks , and J. E. Watson . 2016. “Biodiversity: The Ravages of Guns, Nets and Bulldozers.” Nature 536, no. 7615: 143–145. 27510207 10.1038/536143a

[ece374217-bib-0021] McCarthy, T. , and D. Mallon (Volume Editors). 2016. Snow Leopards: Biodiversity of the World: Conservation From Genes to Landscapes. First ed, 612. Elsevier.

[ece374217-bib-0022] Morrison, J. C. , W. Sechrest , E. Dinerstein , D. S. Wilcove , and J. F. Lamoreux . 2007. “Persistence of Large Mammal Faunas as Indicators of Global Human Impacts.” Journal of Mammalogy 88, no. 6: 1363–1380.

[ece374217-bib-0023] Mu, H. , X. Li , Y. Wen , et al. 2022. “A Global Record of Annual Terrestrial Human Footprint Dataset From 2000 to 2018.” Scientific Data 9, no. 1: 176. 35440581 10.1038/s41597-022-01284-8 PMC9018937

[ece374217-bib-0025] Pulliam, H. R. 1988. “Sources, Sinks, and Population Regulation.” American Naturalist 132, no. 5: 652–661.

[ece374217-bib-0026] Ripple, W. J. , J. A. Estes , R. L. Beschta , et al. 2014. “Status and Ecological Effects of the World's Largest Carnivores.” Science 343, no. 6167: 1,241,484. 10.1126/science.1241484 24408439

[ece374217-bib-0027] Runge, J. P. , M. C. Runge , and J. D. Nichols . 2006. “The Role of Local Populations Within a Landscape Context: Defining and Classifying Sources and Sinks.” American Naturalist 167, no. 6: 925–938. 10.1086/503531 16615034

[ece374217-bib-0028] Soberón, J. 2007. “Grinnellian and Eltonian Niches and Geographic Distributions of Species.” Ecology Letters 10, no. 12: 1115–1123. 17850335 10.1111/j.1461-0248.2007.01107.x

[ece374217-bib-0029] Symes, W. S. , D. P. Edwards , J. Miettinen , F. E. Rheindt , and L. R. Carrasco . 2018. “Combined Impacts of Deforestation and Wildlife Trade on Tropical Biodiversity Are Severely Underestimated.” Nature Communications 9, no. 1: 4052. 10.1038/s41467-018-06579-2 PMC6170487 30283038

[ece374217-bib-0030] Torres‐Romero, E. J. , T. M. Eppley , W. J. Ripple , et al. 2025. “Global Scale Assessment of the Human‐Induced Extinction Crisis of Terrestrial Carnivores.” Science Advances 11, no. 29: eadq2853. 40668902 10.1126/sciadv.adq2853 PMC12266096

[ece374217-bib-0031] Torres‐Romero, E. J. , A. J. Giordano , G. Ceballos , and J. V. López‐Bao . 2020. “Reducing the Sixth Mass Extinction: Understanding the Value of Human‐Altered Landscapes to the Conservation of the World's Largest Terrestrial Mammals.” Biological Conservation 249: 108706.

[ece374217-bib-0033] Venter, O. , E. W. Sanderson , A. Magrach , et al. 2016. “Sixteen Years of Change in the Global Terrestrial Human Footprint and Implications for Biodiversity Conservation.” Nature Communications 7, no. 1: 12,558. 10.1038/ncomms12558 PMC4996975 27552116

[ece374217-bib-0034] Watson, J. E. , D. F. Shanahan , M. Di Marco , et al. 2016. “Catastrophic Declines in Wilderness Areas Undermine Global Environment Targets.” Current Biology 26, no. 21: 2929–2934. 27618267 10.1016/j.cub.2016.08.049

[ece374217-bib-0035] Wolf, C. , and W. J. Ripple . 2017. “Range Contractions of the World's Large Carnivores.” Royal Society Open Science 4, no. 7: 170052. 28791136 10.1098/rsos.170052 PMC5541531

[ece374217-bib-0036] Wood, S. , and M. S. Wood . 2015. “Package ‘mgcv’.” R Package Version, 1(29), 729.

[ece374217-bib-0037] Woodroffe, R. 2000. “Predators and People: Using Human Densities to Interpret Declines of Large Carnivores.” Animal Conservation 3, no. 2: 165–173.

