# Supplementary material for: Human Pressure Structures Range Contraction and Recovery Potential of the Snow Leopard
Source: Ecol Evol. 2026 Sep 2;16(9):e74217. doi: 10.1002/ece3.74217 (PMC13538952; doi:10.1002/ece3.74217)
Supplement: Supplementary file 1 [file ECE3-16-e74217-s001.docx]

**Supplementary Information**

**FIGURES**

**FIGURE S1. Distribution of Human Footprint values across historical, lost, extant and possibly extant range states**. Violin plots illustrate the full distribution of Human Footprint values, whereas embedded boxplots show the median and interquartile range.
